# Cognitive and psychiatric changes as first clinical presentation in Sneddon syndrome

**DOI:** 10.1590/1980-57642018dn12-020016

**Published:** 2018

**Authors:** Giorgio Fabiani, Raul Martins, Gelson Luis Koppe, Zeferino Demartini, Luana Antunes Maranha Gatto

**Affiliations:** 1 Neurologist of Hospital das Nações, Curitiba, PR, Brazil.; 2 Radiologist of CETAC - Diagnóstico por Imagem, Curitiba, PR, Brazil.; 3 Interventional Neuroradiologist of Hospital das Nações, Curitiba, PR, Brazil.; 4 Neurosurgeon and Interventional Neuroradiologist of Hospital das Nações, Curitiba, PR, Brazil.

**Keywords:** Sneddon syndrome, central nervous system vasculitis, presenile dementia, vascular dementia, antiphospholipid syndrome, síndrome de Sneddon, vasculite do sistema nervoso central, demência pré-senil tipo Alzheimer, demência vascular, síndrome antifosfolipídica

## Abstract

Sneddon syndrome (SS) is a rare progressive non-inflammatory thrombotic vasculopathy affecting small/medium-sized blood vessels of unknown origin. It is strongly associated with the presence of antiphospholipid antibodies (AA). The presence of livedo reticularis and cerebrovascular disease are hallmark features. The condition is far more common in young women. We report a case of SS in a 43 year-old male with a two-year history of progressive cognitive impairment consistent with dementia syndrome, and major personality changes, besides livedo reticularis and cerebral angiographic pattern of vasculitis. AA were borderline. The recognition of skin blemishes that precede strokes should raise the hypothesis of SS. AA are elevated in more than half of cases, but their role in the pathogenesis or association of positive antibodies and SS remains unclear. Dementia syndrome in young patients should be extensively investigated to rule out reversible situations. Typical skin findings, MRI and angiography may aid diagnosis.

## RESUMO

A síndrome de Sneddon (SS) é uma vasculopatia trombótica não inflamatória progressiva rara que afeta os vasos sanguíneos de pequeno e médio tamanho com origem desconhecida. Está fortemente associada à presença de anticorpos antifosfolipídios (AA). A presença de livedo reticularis e doença cerebrovascular são a marca registrada. É muito mais comum em mulheres jovens. Relatamos um caso de SS em um homem de 43 anos de idade com dois anos de história de comprometimento cognitivo progressivo compatível com síndrome demencial e mudanças graves de personalidade, além de livedo reticular e padrão angiográfico cerebral de vasculite. AA eram limítrofes. O reconhecimento das manchas da pele que precedem eventos isquêmicos cerebrovasculares deve reforçar a hipótese de SS. Os AA são elevados em mais da metade dos casos, mas seu papel na patogênese ou associação de anticorpos positivos e SS permanece obscuro. A síndrome demencial em pacientes jovens deve ser amplamente investigada para se descartarem situações reversíveis. Achados típicos da pele, ressonância magnética e angiografia podem ajudar no diagnóstico.

In 1965, Sneddon described^1^ six patients with a new syndrome whose main symptoms included multiple episodes of lacunar subcortical, ischemic infarcts and widespread livedo eruption. Today known as Sneddon’s syndrome (SS), it is a rare non-inflammatory thrombotic vasculopathy, characterized by cerebrovascular disease and typical skin lesions, the livedo reticularis.^1^^-^ 6 The Orpha number for SS is ORPHA820.^2^ It has a chronic progressive course,^2^^,^ 3 and is strongly associated with the presence of antiphospholipid antibodies.^3^ The incidence of cases is 4 per 1 million per year, and a mortality rate of 9.5% was reported in a mean observation period of 6.2 years.^2^ SS is far more common in young women between 20 and 42 years of age^2^^-^ 5 and has a wide spectrum of physical, neurologic, and laboratory findings. The neurological signs include severe cognitive impairment or dementia syndrome and psychiatric changes.^2^^,^ 3 SS can be associated with valvulopathy, a history of spontaneous abortion, renal involvement, vascular dementia, systemic hypertension, acrocyanosis, Raynaud’s phenomenon, secondary headaches, venous thrombosis and seizures.^2^^-^ 5

Antiphospholipid antibodies can be found at a highly variable frequency, and SS may be associated with antiphospholipid syndrome. There are three described forms of SS: primary, autoimmune with antiphospholipid antibodies or mixed type (coexisting systemic lupus erythematosus or lupus-like disease), and a thrombophilic form.^3^

For brain investigation, magnetic resonance imaging (MRI) and cerebral angiography are mandatory.^2^^-^ 4 There is no gold standard treatment, although antiplatelet therapy or anticoagulation are the most recommended options.^3^^-^ 5

The objective of this report is to provide a highly detailed description of SS with dementia and personality changes as the main features, after several silent and unnoticed brain strokes. The patient, his family and many other assisting doctors overlooked the typical skin changes and we believe the cognitive decline could have been reduced with early diagnosis.

## CASE REPORT

**Figure 1:**
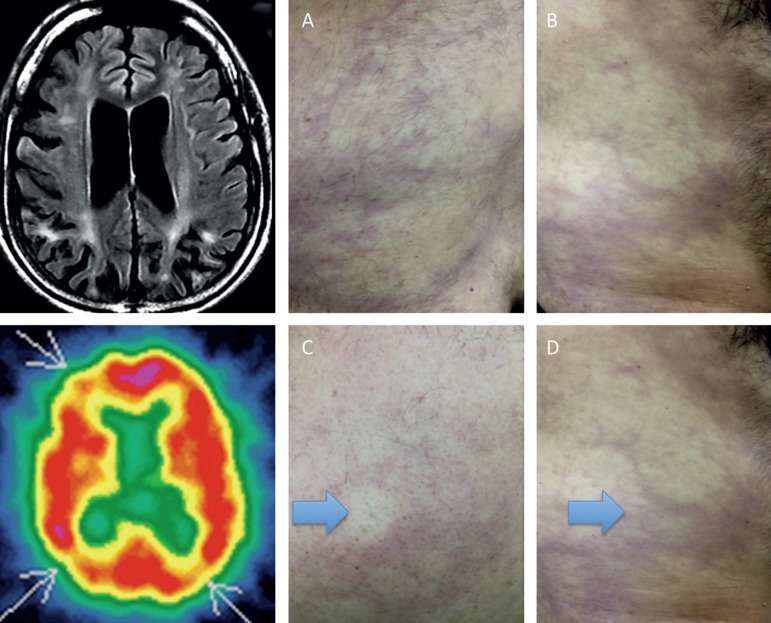
Right side with blue arrows – Livedo reticularis diffusely spread throughout the thoracic and abdominal circumference (A,B,C and D), with rapid return with finger pressure (C with blue arrow). Upper left. Axial Flair Brain MRI shows multiple areas of infarction in cortico-subcortical regions and in white matter, and diffuse brain atrophy and perfusion with 99mTc-ECD – Brain SPECT shows marked diffuse hypoperfusion in parietal and frontal lobe areas (arrows).

The patient is a 43-year-old man, Latin-American, with degree-level education. He attended the medical consultation after his wife noted a two-year history of progressive forgetfulness, mental confusion, disorientation, difficulty finding the right words, changes in mood (basically from being shy to outgoing). He also lost his job for poor performance and was rejected by his friends as a consequence of his new outgoing personality. Concomitantly, he started experiencing sleep changes and apathy, together with anxiety symptoms. Initially he was treated as suffering from major depression, and later as type II bipolar disorder. The treatments failed to change his behavior. The physical examination was completely normal, including pulmonary and cardiac auscultation (normal echocardiogram), except for multiple skin blemishes, mainly on the trunk. Neither he nor his wife recognized his skin changes as abnormal. The blemishes were spread all around his trunk and belly, and changed rapidly under finger pressure (Figure 1 - right side with blue arrows). The patient was submitted to neuropsychological tests and we provide here a brief overview of the results: he scored 23 points on the Mini-Mental Status Examination and only 15 points on the MOCA test. He scored 30 points on the HAM-D scale and 20 on the Hamilton anxiety scale. The patient was CDR 1 and FAST stage was 5 (moderate disease). In general, the patient performed poorly on all neuropsychological tests, with moderate-to-severe decline in cognitive functions, including declarative memory, attention, and poor language and executive function results. He also presented many emotional disturbances that were negatively affecting his life. No further neurological signs were found. Lastly, neither the patient nor his wife described any stroke-like episodes. He reported no family history of livedo reticularis, stroke, vasculitis, or SS.

Laboratory findings showed undetermined anticardiolipin antibodies, besides the weak presence of lupic anticoagulant. Laboratory studies for venereal disease, human immunodeficiency virus and hepatitis B and C tests, protein C and protein S, C-reactive protein, sedimentation rate, rheumatoid factor, antinuclear factor and other rheumatic testing were completely normal.

The brain MRI (Figure 1) - upper left showed multiple areas of signal changes on cortico-subcortical transition and in the deep periventricular white matter with gadolinium enhancement, consistent with brain ischemia. Imaging also disclosed diffuse cerebral atrophy disproportionate for his age. Brain Perfusion with 99mTc - ECD brain SPECT showed accentuated diffuse hypoperfusion in parietal and frontal areas (Figure 1) - lower left.

**Figure 2:**
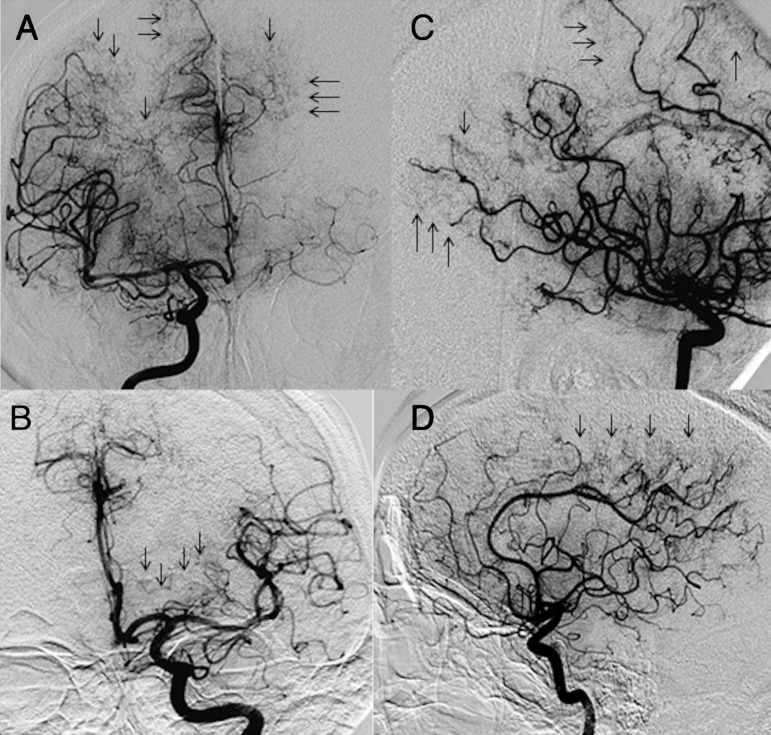
Cerebral angiography (late arterial phase) with multifocal narrowing in the distal cortical branches, parietal irregularities and parenchymal filling with blush in the corresponding watershed areas. **[A]** Right internal carotid artery (ICA) on anteroposterior (AP) view. **[B]** Right ICA on lateral view. **[C]** Left ICA on AP view. **[D]** Left ICA on lateral view.

Cerebral angiography (Figure 2) revealed slowed distal blood flow of cerebral arteries with decreased vessel diameter and parietal irregularities suggestive of vasculitis. The venous phase was normal, and there was no vascular malformation. The anterior, middle and posterior cerebral arteries in their respective distal segments showed segmental lesions with reduced caliber associated with tortuosity and slowed flow, characterizing a late blush in the territory of the watershed zone. The patient was discharged from the hospital with a diagnosis of SS and the following treatment: acetylsalicylic acid, corticoids and oral anticoagulants.

We obtained the approval of the institution’s ethics committee and the patient’s informed consent form.

## DISCUSSION

Another syndrome, called Divry van Bogaert Syndrome (DBS), is a familial juvenile-onset disorder characterized by livedo racemosa, white matter disease, dementia, epilepsy and angiographic finding of “cerebral angiomatosis”, raising the question as to whether DBS and SS can be considered different entities or indeed different features of the same syndrome.^5^ Our patient more closely matched the criteria for the diagnosis of SS.

The cognitive decline is explained by vascular dementia and due to significant atrophy. Cerebral atrophy is described in SS as a progressive complication due to involvement of small arteries.^2^^,^ 4 ^,^ 5 ^,^ 7

The antiphospholipid antibodies of our patient were not conclusive to establish the diagnosis of SS or another disease. Although the pathogenesis of SS with the presence of antiphospholipid antibodies may be explained in a similar manner to the pathogenesis of antiphospholipid syndrome, the significance of the presence of these antibodies in both syndromes and the relationship between antiphospholipid syndrome and SS are unclear.^2^^-^ 5 Studies of patients with SS reveal elevated antiphospholipid antibody levels in around 57% of patients (range 0-85%)^2^^,^ 3 matched with normal controls. However, in some patients these antibodies are consistently absent, indicating that SS may be a distinct entity or perhaps a group of different disorders, given there are clinical differences in patients with or without antiphospholipid antibodies.^3^

Livedo reticularis often precedes the cerebrovascular events, whose onset usually occurs before the age of 45 years.^2^^,^ 5 The cerebrovascular events consist of ischemic strokes or transient ischemic attacks, which affect mainly medium-sized arteries and are seen particularly in the territory of the middle and posterior cerebral artery.^2^^-^ 6 The patient’s cerebral angiogram showed diffuse distal multifocal narrowing, and a luxury perfusion mainly in the watershed zones between the anterior and middle cerebral arteries.

The finding of vasculitis on cerebral angiography was the last piece in the puzzle of this case. Had this been the sole finding, it would have posed a major diagnostic challenge. Cerebral angiography is abnormal in up to 75% of patients with SS. The most common abnormality is an obliterating non-inflammatory arteriopathy, with stenosis and/or occlusion of intracranial vessels (Figure 2). In our case, cerebral DSA revealed decreases in calibration, contour irregularities, and blockages mainly in anterior circulation.

In conclusion, we reported a highly detailed description of SS with dementia and personality changes as the main features, after several silent and unnoticed brain strokes.

The patient, his family and many other assisting doctors overlooked the typical skin changes and we believe the cognitive decline could have been reduced with early diagnosis. Since the outset, the main complaints were cognitive, as we demonstrated in the neuropsychological results. The cognitive changes were certainly secondary to brain changes, as illustrated by the MRI and SPECT findings.

We would like to point out the importance of an extensive diagnostic panel when dealing with young patients exhibiting dementia symptoms.

This case illustrates the importance and severity of SS as well as the wide range of differential diagnoses. Young patients whose neuroimaging exams show strokes should be followed by a neurologist with vascular expertise and submitted to rheumatologic, serologic and thrombophilic tests, besides angiography and MRI. We would like to reinforce the importance of a thorough clinical and physical examination to disclose skin changes, mandatory in SS diagnosis.

The literature review underscores the need for a detailed work-up to better clarify the relationship between antiphospholipid antibodies and SS.
